# mRNA Expression of EgCHI1, EgCHI2, and EgCHI3 in Oil Palm Leaves (*Elaeis guineesis* Jacq.) after Treatment with *Ganoderma boninense* Pat. and *Trichoderma harzianum* Rifai

**DOI:** 10.1100/2012/647504

**Published:** 2012-08-02

**Authors:** Laila Naher, Soon Guan Tan, Chai Ling Ho, Umi Kalsom Yusuf, Siti Hazar Ahmad, Faridah Abdullah

**Affiliations:** ^1^Department of Biology, Faculty of Science, Universiti Putra Malaysia, Selangor, 43400 Serdang, Malaysia; ^2^Department of Cell and Molecular Biology, Faculty of Biotechnology and Biomolecular Sciences, Universiti Putra Malaysia, Selangor, 43400 Serdang, Malaysia; ^3^Institute of Tropical Agriculture, Universiti Putra Malaysia, Selangor, 43400 Serdang, Malaysia; ^4^Department of Crop Science, Faculty of Agriculture, Universiti Putra Malaysia, Selangor, 43400 Serdang, Malaysia

## Abstract

*Background*. Basal stem rot (BSR) disease caused by the fungus *Ganoderma boninense* is the most serious disease affecting the oil palm; this is because the disease escapes the early disease detection. The biocontrol agent *Trichoderma harzianum* can protect the disease only at the early stage of the disease. In the present study, the expression levels of three oil palm (*Elaeis guineensis* Jacq.) chitinases encoding EgCHI1, EgCHI2, and EgCHI3 at 2, 5, and 8 weeks inoculation were measured in oil palm leaves from plants treated with *G. boninense * or *T. harzianum* alone or both. *Methods*. The five-month-old oil palm seedlings were treated with Gano-wood blocks inoculum and trichomulch. Expression of EgCHI1, EgCHI2, and EgCHI3 in treated leaves tissue was determined by real-time PCR. *Results*. Oil palm chitinases were not strongly expressed in oil palm leaves of plants treated with *G. boninense* alone compared to other treatments. Throughout the 8-week experiment, expression of EgCHI1 increased more than 3-fold in leaves of plants treated with *T. harzianum* and *G. boninense* when compared to those of control and other treated plants. *Conclusion*. The data illustrated that chitinase cDNA expression varied depending on tissue and the type of treatment.

## 1. Introduction

The oil palm (*Elaeis guineensis *Jacq.) is an important economic crop that produces two types of oils: palm oil from the fibrous mesocarp and kernel oil from the seeds. Currently, the oil palm industry is under threat from a fungal disease called basal stem rot (BSR), which is caused by the fungus *Ganoderma boninense *Pat. [1, 2]. Other fungal diseases, such as vascular wilt (caused by *Fusarium oxysporum* f.sp. *Elaeidis*) and sudden wilt (caused by *Phytomonas staheli* McGhee) [1], also affected the oil palm but BSR is by far the most serious among them; it causes tree loss in palm stands and subsequent loss in yield of palm oil [1, 3]. The disease escapes early detection: by the time fruiting bodies are detected, the disease is too advanced to response to any chemical treatments.

The use of the fungus *Trichoderma* spp. as a biocontrol agent for controlling plant disease was first recognized in the early 1930s [4]. Subsequently, many studies have shown that *Trichoderma* spp. are the most effective biocontrol agents for managing plant disease. *Trichoderma* controls the pathogen via a mycoparasitism process in which it grows towards the pathogenic fungi, coils around them, and secretes cell wall degrading enzymes that limit their growth [5]. An *in vitro* study showed that *Trichoderma* produced trichodermin and antimycotin, which are compounds that inhibited the growth of *Rhizoctonia solani* [6]. Harman et al. [7] proposed a mechanism of disease control that involves the release of cell wall degrading enzymes from *Trichoderma* which activates the expression of genes involved in the plant defence system. *Trichoderma* sp. has been proven to be highly effective for controlling *Ganoderma boninense*/BSR disease in oil palms but only at the early stage of slightly infected palms [8–10]. Therefore, to date, there is no adequate control measure to control BSR improvements of the oil palm defence system against *G. boninense *is the alternative option.

Plants use various defence mechanisms during plant-microbe interactions, including the strengthening of physical barriers (e.g., lignin and cellulose), synthesis of antimicrobial compounds (phytoalexins), and synthesis of pathogenesis-related (PR) proteins. Chitinases are PR proteins that hydrolize the *β*-1, 4 glycosidic bond in chitin, which is found in most fungal cell walls and is a common constituent of insect cuticles and crustacean shells [11, 12]. Moreover, the breakdown products of chitin may serve as elicitors of the plant defence reaction [13]. Chitinase also expressed at low levels under normal conditions during plant developments [14]. Thus, the physiological expression of chitinases in plants can be both constitutive and induced by biotic or abiotic stresses or induced in pathogen infection [15–18].

The plant chitinases are divided into seven classes (I through VII) based on their structural properties and amino acid sequence similarities [19]. The current view is that not all chitinases are induced in response to pathogen attack: instead, only specific chitinases are stimulated by a particular pathogen. For example, class I chitinase from tobacco showed antifungal activity against *Fusarium solani* germlings, whereas class II chitinases showed only slight antifungal activity when used with *β*-1,3 glucanase [20]. In Norway spruce (*Picea abies* L. Karst.) plants (clones 409 and 589) when expression of chitinase classes I, II, and IV was monitored after wounding and infection by the fungus *Heterobasidion annosum* Fr., maximum transcript levels for classes II and IV were found in both clones compared to class I [21]. Apart from their role in pathogen defence, chitinases also have a role in symbiotic or biocontrol agent interaction in plant. Our previous study showed that chitinases expression was high in oil palm root tissues when *G. boninense* infection first appeared in root tissues but the expressions declined during the development of the disease while in *T. harzianum* alone or together with *G*. *boninense* treated oil palm plants; chitinases expression remained upregulated at the end of the experiment in oil palm root tissues [22]. However, some chitinases are developmentally regulated or induced by specific organs. Thus, the purpose of this present study was to investigate the chitinases expression in oil palm leaf tissues treated with the pathogen *G. boninense* Pat. and the biocontrol agent *T. harzianum* Rifai either alone or in combination.

In this study, three oil palm chitinase cDNAs, previously isolated from oil palm encoding EgCHI1 (GenBank accession number ADC55619) which matched with plant chitinase, chitinase class I from *Arabidopsis thaliana* (AAF29391.1), EgCHI2 (HQ831445) which matched with plant chitinase, chitinase class II from *Fragaria *x* Ananassa* (AAF00131.1), and EgCHI3 (HQ831446) which matched with plant chitinase chitinase class III from *Bambusa Oldham* (ABW75909.1) (Naher et al. [22]), were used to investigate the expression levels of chitinases in the leaves of oil palms artificially inoculated with* G. boninense*. Whether the presence of the BSR biocontrol agent *T. harzianum* affected chitinase expression was also evaluated.

## 2. Materials and Methods

### 2.1. Preparation of Plant Treatment Materials

The cultures of seven-day-old *G. boninense* and *T. harzianum* were used for prepared Gano-wood blocks and trichomulch, respectively. Freshly cut rubber wood blocks were used carrier for Gano-wood blocks and palm-pressed mesocarp fibers were used carrier for trichomulch. The preparation of plant treatment materials consisting of Gano-wood blocks and Tricho-mulch have been described previously [22].

### 2.2. Plant Treatments

The experiments were conducted in a glass greenhouse over 8-week period. The 5-month-old oil palm seedlings used in the experiment were provided by Sime Darby Seeds & Agricultural Services Sdn Bhd (Banting, Selangor, Malaysia). Each of four treatments (control, *G. boninense* Pat., *T. harzianum* Rifai, and *G. boninense* + *T. harzianum*) was replicated three times.

The control treatment consisted of an oil palm plant in a garden pot. The artificial inoculation of oil palms with *G. boninense* Pat. followed by Naher et al. [22]. Briefly, a Gano-wood block was placed in direct contact with the roots of a plant in a garden pot and then covered with soil. For *Trichoderma-*inoculated treatments, 600 g of Trichomulch were placed on the surface of the soil. Plants in the *G. boninense* + *T. harzianum* group were treated with both a Gano-wood block and Trichomulch. The seedlings were watered twice daily using tap water.

In this study, the gene expressions of oil palm chitinases at the early stage of the plant-microbe interaction were investigated. *Ganoderma* is a slow-growing fungus that requires more than 1 week to develop mycelia on the root surface. Thus, the first samples were collected at 2 and the plants then sampled again at 5 and 8. Control and treated leaves were excised using a clean scissors, dried with paper towels, and then weighed. Then, they were wrapped in aluminium foil (1 g/pack) for RNA extraction. The leaf tissues were frozen immediately in liquid nitrogen and stored at −80°C.

### 2.3. RNA Extraction

Total RNA was extracted from treated and untreated leaf tissues using a modified cetyl trimethyl ammonium bromide (CTAB) method [23]. RNA extraction from oil palm was previously described in detail [22]. Briefly, 1 g of tissue was ground in liquid nitrogen into very fine powder which was immediately transferred to a 50 mL polypropylene tube containing 15 mL of CTAB extraction buffer. Next, an equal volume of chloroform : isoamyl alcohol (C : I) (24 : 1) was added to the tube and then centrifuged at 12, 857 g for 15 min at 4°C. The upper layer was carefully transferred to a new 50 mL polypropylene and 15 mL of phenol:chloroform:isoamyl alcohol (P :C: I) (25 : 24 : 1) was added. Centrifugation was performed using the conditions as described above. The final supernatant was adjusted to a final concentration of 2 M LiCl for incubation at 4°C overnight.

After overnight incubation, the homogenate was centrifuged at 12,857 g for 30 min at 4°C. The pellet was dissolved in 5 mL of diethylpyrocarbonate-(DEPC) treated water, and then an equal volume of C : I was added and centrifuged at 12,857 g for 15 min at 4°C. The supernatant was transferred to a new tube and the RNA was precipitated by adding 0.1 volume of 3 M sodium acetate pH 5.2 and 2.5 volumes of 100% ethanol, followed by incubation at −80°C overnight. After centrifugation, the resulting pellet was washed with 70% (v/v) ethanol. The pellet was air dried and resuspended in DEPC-treated water. The RNA purity was examined using a spectrophotometer at 230, 260, and 280 nm and integrity of RNA was examined using 1% denaturing formaldehyde agarose gel electrophoresis [24]. Then RNA was treated with DNase I (Qiagen, USA) according to manufacturer's instructions.

### 2.4. Designing of cDNA Primers

To measure the expression of chitinases in oil palm, the primers were designed using Primer 3 software version 0.4.0 based on the 3′untranslated region (UTR) of oil palm chitinase cDNAs as already isolated in our previous study [22]. The following primers were used for real-time RT : PCR : EgCHI1-F, 5′-GCT GTC CAT CAA TTG GAT CCT C-3′ and EgCHI1-R, 5′-CTT TAC TGG CGT GGT TCG AGT-3′; EgCHI2-F, 5′-TCG GAA TTT TTG GTC CTT TTT-3′ and EgCHI2-R, 5′-GTT TAG GGC TTG ATC AGC- 3′; and EgCHI3-F, 5′-TGT CAT ATC ATC TCC AGT TCC AG-3′ and EgCHI3-R, 5′–GAG TTT GTA CGG TTG CCC CTG-3′; actin-F, 5′-CCC ACC TGA ACG GAA ATA CA-3′ and actin-R, 5′-CGG ATG GCA CCT CAG TCT TA-3′. The actin gene (Genbank accession number EL691466) was used as an endogenous control.

### 2.5. cDNA Translation and Reverse Transcriptase (RT-)PCR

To conduct the chitinase expression analysis, total RNA was translated into cDNA. Equal amounts of DNase-treated RNA (1 *μ*g) of control and treated samples were converted into cDNA using the quantitative reverse transcript cDNA synthesis kit following the manufacturer's instructions (Qiagen, USA). Briefly, 1 *μ*g of total RNA and 2 *μ*L 7X gDNA wipe buffer (provided in the kit) were transferred into a clean PCR tube, followed by the addition of DEPC-treated water to a total volume of 14 *μ*L. The mixture was then incubated at 42°C for 2 min and chilled on ice quickly. The remaining components of the kit were added to the reaction mixture, followed by 4 *μ*L of 5X quantiscript RT buffer, 1 *μ*L of RT primer mix, and 1 *μ*L of reverse transcriptase enzyme; the mixture was incubated at 42°C for 30 min. Finally, the reaction was heated at 95°C for 3 min to terminate the cDNA synthesis reaction, and the cDNA was stored at −20°C.

### 2.6. Real-Time RT-PCR

Real-time RT-PCR was performed using the Bio-Rad iQ5 real-time PCR system (Bio-Rad, USA). Equal amounts of RNA (1 *μ*g) extracted from control and treated oil palm leaves samples were converted into cDNA by using the quantitative reverse transcript cDNA synthesis kit (Qiagen, USA) following the manufacturer's instructions. Real-time RT-PCR was performed on EgCHI1, EgCHI2, and EgCHI3 together with actin in three replicates in one 96-well plate and PCR conditions were as follows: 1 cycle of 95°C for 10 min followed by 40 cycles of 95°C for 30 s, 60°C for 1 min, and 72°C for 1 min. The annealing temperature for all targets and the endogenous control was 60°C.

### 2.7. Real-Time PCR Analysis

Real-time PCR was used to analyze the mRNA expression level of each transcript encoding EgCHI1, EgCHI2, and EgCHI3 in oil palms leaves in interaction with *G. boninense* Pat. and *T. harzianum *Rifai. The relative expression of each transcript was calculated *by* the ΔΔC*_T_* method [25] using iQ5 software (Bio-Rad); the expression levels of EgCHI1, EgCHI2, and EgCHI3 were estimated after being normalized to the endogenous control gene and the significant expression levels were considered if the standard error ≤0.5.

## 3. Results and Discussion

BSR which is caused by the fungus *G. boninense* Pat. is a serious disease that affects the oil palm and is a major threat to the oil palm industry. To date, there is no adequate measure to control this disease, and researchers are looking for ways to improve the oil palm's defence system against *G. boninense *Pat. Plant chitinases are PR proteins that belong to the repertoire of plant defence mechanisms that are believed to constitute the early defence response in plants. Generally, chitinase induction is considered to be part of the nonspecific defence reaction initiated in a plant after pathogen attack or exposure to physical, chemical, or environmental stresses [26]. Thus, the plant chitinases may be involved in the oil palm's reaction to infection by *G. boninense *Pat.

The goal of this study was to investigate the potential role of chitinase mRNA expression in oil palms infected by *G. boninense* Pat. as well as in samples treated by *T. harzianum*, which a biocontrol agent is used to combat BSR disease. Prior to running real-time RT-PCR for the expression study, primers of EgCHI1, EgCHI2, and EgCHI3, and an endogenous control (actin) were optimized for annealing temperature. The annealing temperature of all of the primers optimized at 60°C. Afterwards, real-time PCR was performed for the target transcripts. The PCR efficiencies of all targets and the endogenous control were approximately equal (91-92%).

**Figure 1:**
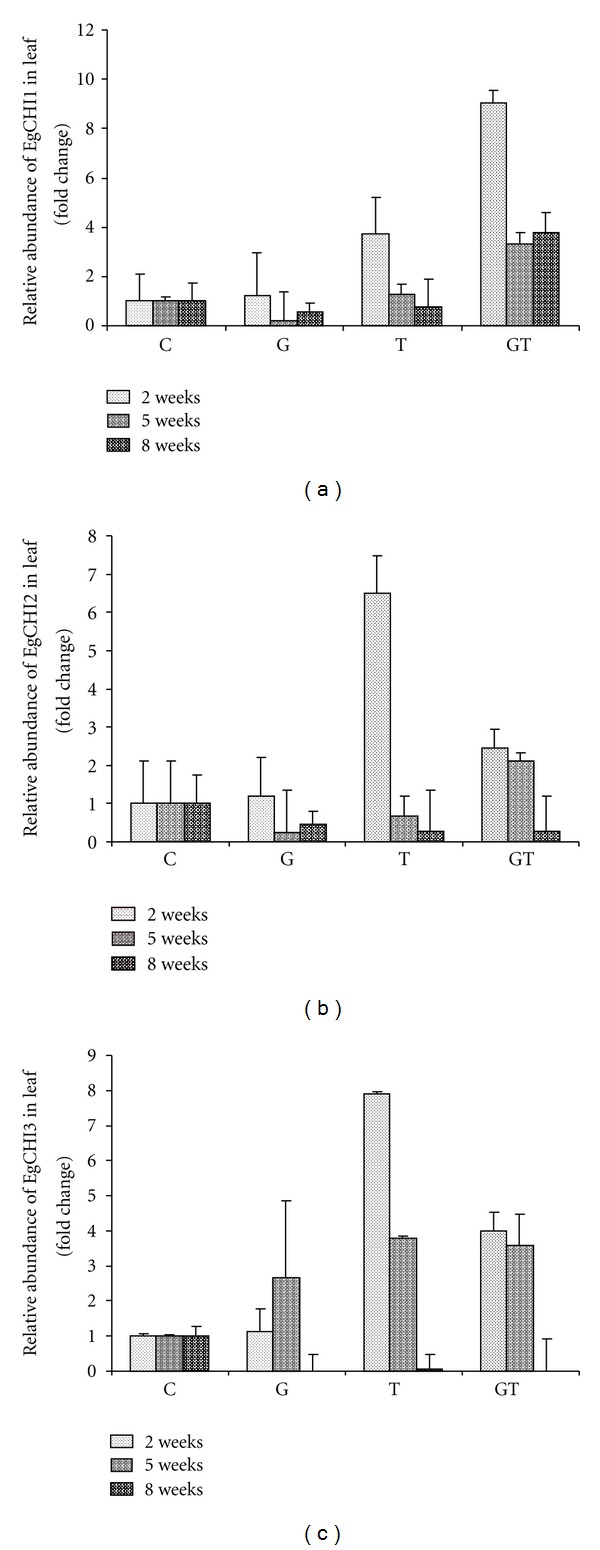
Relatives abundances of EgCHI1, EgCHI2, and EgCHI3 at various time points in oil palm leaf tissues, inoculated with *G. boninense* and *T. harzianum* either alone or together. To compare the levels of the transcripts among the treatments, the value of the control plant transcripts was set at 1 and the data for the treatments were then normalized to this value. Error bars indicate standard errors. The expression level was considered significant if the standard errors ≤ 0.5. C = Control, G = *Ganoderma*, T = *Trichoderma*, GT = *Ganoderma* +*Trichoderma. *

Figures 1(a), 1(b), and 1(c) show the relative expression levels of EgCHI1, EgCHI2, and EgCHI3, respectively, in leaves in response to inoculation with *G. boninense* Pat. and *T. harzianum* Rifai alone or in combination at different time points compared with that of the control plants. In *G. boninense* alone treated plants, no significant upregulation (SE > 0.5) in expression of any of the transcripts was observed at any time points. It was reported from previous study that the pathogenesis-related (PR) protein chitinase was elicited in plants during early response to the pathogen attack [27, 28]. However, none of the oil palm chitinases studied was strongly induced against* G. boninense*. It could be that the earliest time point of this study was too late to reflect gene expression against the pathogen or that oil palm chitinases might serve as a local or as an organ-specific defence mechanism, as the chitinase expression results from oil palm leaves found in this study differed from those detected in root tissues of plants treated in the same way [22]. This result was in contrast to that of a previous study [22] in which at 5 weeks after the disease was first observed only in root tissue, all the transcripts were upregulated.

In the plants treated with *T. harzianum* alone, significant upregulation was detected only for expression of EgCHI3: a 7.9-fold and 3.8-fold increased at 2 and 5 wpi (Figure 1(c)), respectively. In the *T. harzianum* + *G. boninense* treatment, the expression of EgCHI1 (Figure 1(a)) was dramatically increased (9.03-fold, 3.3-fold and 3.8-fold at 2, 5, and 8 wpi, resp.) and that of EgCHI2 (Figure 1(b)) was also up-regulated (2.44-fold and 2.1-fold at 5 and 8 wpi, resp.). In the same treatment, the EgCHI3 expression (Figure 1(c)) was up-regulated (4-fold and 3.57-fold) at 2 wpi and 5 wpi, respectively, and at week 8 the EgCHI3 expression was very low in the presence of all fungi-treated oil palm plants. A similar result was observed in grapevine (*Vitis vinifera* L.) infected with *Plasmopara viticola*: chitinase class III was expressed only twice in the early stages (days 2 and 6) of a 10-day experiment, but in healthy leaves, the expression was low at day 2 and increased at the late stage of 6 to 8 days [29]. In another study, chitinase class III was not expressed in the leaves of *Vitis vinifera* infected by the pathogen *Botrytis cinerea*, but class I was expressed; moreover, in the control plant both classes were constitutively expressed [30]. These authors suggested that the timing of sporulation of the fungi and the nature of the infection were reflected in the plant's gene expression. Overall, the expression data showed that oil palm chitinases expression was higher when plants were treated with *Trichoderma* and it might be that *Trichoderma* helped to induce chitinases in plants. Shores and Harman [31] found that chitinase activity was higher in plant from *Trichoderma*-treated seeds than from untreated seeds. Hence, they suggested that *Trichoderma*-treated plants expressed chitinase as defence mechanism to be more resistance to disease.

In conclusion, chitinase expression was not significantly increased in *Ganoderma*-alone-treated plants. In terms of susceptibility, plant chitinases that are expressed at low levels under normal conditions may not be strong enough to ward off fungal pathogen growth [32, 33]. Since not all classes of chitinases were investigated in this study, the roles of other chitinase classes in oil palm during pathogen attack deserved further attention. Our data also illustrated that the presence of *Trichoderma* might be involved in inducing chitinase expression especially EgCHI1 in oil palms to enhance defence mechanism.
